# Case of Daniel Brown, Seaman, Who Was Struck Both Dumb and Blind with Lightning, On-Board the Said Ship, While at Sea, on Friday, the 21st of February, 1799, Being Then a Few Leagues off the Eddystone Light-House

**Published:** 1822-05

**Authors:** James Godfrey

**Affiliations:** Surgeon of the Cambrian.


### PHYSIOLOGICAL MEDICINE.

THE singularity of many of the circumstances attending the following relation, and its indubitable authenticity, have induced us to give it a place in our Journal, although its date be not recent. Indeed, we share in the opinion of many practitioners, that facts are independent of dates, provided they be intrinsically valuable as to instruction, interesting as to novelty, and credible from the nature of the authority on which they are related. Of the correctness of those which our readers are about to peruse, we think we can fearlessly answer. The paper was obtained from the most unobjectionable source.

A. B. G.

---

*His Majesty's Ship Cambrian, at Sea.*

ART. V.—*Case of Daniel Brown, Seaman, who was struck both Dumb and Blind with Lightning, on-board the said Ship, while at Sea, on Friday, the 21st of February,* 1799, *being then a few Leagues off the Eddystone Light-house.* Taken from the Journal of Mr. JAMES GODFREY, Surgeon of the Cambrian.—N.B. *There were two other Men killed on the spot, and twelve, more or less, hurt, so as to be rendered unfit for duty for some time.*

SPEAKING of Brown's case, he says:

"I attached myself principally to Daniel Brown, as it was thought we might entertain some hopes of his recovery. He was becoming cold, without any pulsation at the wrist; in a state of stupor, with froth issuing from the mouth. He was immediately stripped of his clothes, and laid between warm blankets; and, by strictly persevering in the means recommended by the Humane Society to be used on these occasions, for more than an hour, a weak pulsation at the wrist was perceived; the colour began to return in his lips and face, and a general warmth to take place over the whole body. Animal heat was thus excited gradually; the pulsation of the temporal and carotid arteries became more frequent; the eyes closed and opened once or twice, with violent convulsions of the head and spine.

"The pulse at this time became very strong and full. He was then bled to the quantity of eight or ten ounces; and a few tea-spoonfuls of warm rum and water given, which he swallowed with difficulty. He then slept soundly for two hours; and, as he was considered beyond danger, left with careful attendants for the night. He slept well; made a moaning noise; but had not spoken to any body, although questions had been asked.

"On visiting him early the next morning, it was found that he had lost all power of elevating the eyelids, and likewise of speech.

"The eyes, on inspection, appeared of their natural size, and without any apparent defect, excepting a small dilatation of both pupils. The eyes, however, were somewhat drawn upwards and inwards, and he did not possess the power of rolling or moving them freely; neither could he, with the utmost efforts, raise, in the smallest degree, either of the upper eyelids. He was entirely deprived of the power of speech, and, in a great manner, of those of deglutition. He was perfectly sensible, and, by signs, notified that he felt great pain of the forehead, through the orbits, and in the throat; which last symptom was much aggravated whenever he attempted to swallow any thing; and the pain that ensued was so very severe, as to prevent his taking that nourishment which his good appetite otherwise prompted him to do.

"In motion, he suffers pain down the course of the spine, and with difficulty moves the lower jaw; the tongue is drawn far back into the mouth, and cannot be moved easily from side to side, and appears much smaller than I ever before observed.

"The head was shaved, and compresses, wet with a cooling lotion, frequently applied; the throat and neck were well rubbed with a strong volatile liniment.

"At first he passed neither urine nor feces; but, towards the evening, he made nearly three pints of water. I became anxious for the bowels being open, and ordered the underwritten:

℞. Calomel, gr. viij.
Cons. Cynos. q. s. M. fiat pil. ij.

These two small pills were put into a small tea-spoonful of tea, and swallowed without much difficulty. They procured three motions.

"In the evening a blister was applied, from the top of one ear across the throat to the other, which has answered the end so far that he takes fluids with much greater ease, and, by what we can learn, finds himself better. The inability to open the eyelids still continues. He now uses this lotion:

℞. Opii pulv. ʒss.

Aq. bullient, ℥vj. M. fiat lotio.

"In consequence of bearing-up for Torbay, an opportunity presented for his removal into the Plymouth Hospital, where he remained more than three months, under the care of the medical gentlemen of that establishment; and from whom I learned his subsequent treatment.

"He was frequently and smartly vomited, and required doses of unusual strength to produce that action on the stomach. Numerous blisters were applied to the forehead and throat, severe strong liniments, and inhaled into the mouth the vapour of ether. He took large quantities of stimulant, tonic, anti-spasmodic, and anti-epileptic medicines; used the pulvis sternutatorius once, which produced such violent sneezing as threw him into a fit; and it may be worthy to remark, that they generally returned whenever the cause of his misfortune, or electricity, was mentioned to him.

"Under trial of these various medicines, the following relief was obtained:—He could swallow without any difficulty, and could raise both eyelids, though imperfectly; recovered the sight of one eye, could make a noise and whistle, but not articulate one single word; the pain about the spine and throat went off.

"In June, at his particular request, he was discharged from the hospital. On his way to London, an opportunity presented of my seeing him at Portsmouth, on-board the Cambrian. At this time, to the credit of his worthy shipmates, a subscription was set on foot among themselves, which furnished him with a considerable sum; and it is but justice to them, in this place, to relate, that, immediately after the accident, they nobly came forward, in the same manner, with a subscription for the widow of one of the unfortunate men, who was killed on the spot, and, with the officers' contribution, presented her with a purse of fifty pounds; Captain Legge presenting her himself with a twenty-pound bill.

"Brown was then forwarded to London, and placed, by Captain Legge, under the care of a gentleman of eminence, from whom he derived some benefit; but his complete recovery was as truly miraculous as the cause of his misfortune had been awful and alarming.

"A cook's warrant had been procured for him to some ship at Deptford; when, on his way from town, where he had been to be electrified, he was, for the first time since his accident, overtaken in a thunder-storm, with lightning, &c. He was so much shocked and alarmed, that he run into the first house he found open, and from thence into the cellar, where he remained in the most agonizing state until the storm subsided; the people of the house in vain attempting his removal.

"When he had in some degree recovered, and found the storm was over, he ventured into an upper room, took his Bible out of his pocket, and began to read; and, looking round with the utmost astonishment, he thought some person in the room was reading the same words to him: he stopped, read again, and still made the same remark, and then, to his inexpressible joy, found that his own voice had returned.

"He had been so long unaccustomed to it, that he took his pen and paper out to make the bystanders acquainted that he could again speak. I have since this time received a letter from him, and he remains in the most perfect health.

"It appears that the defect of sight in this patient was owing to a paralysis of the optic nerves, in consequence of the severe shock sustained by the lightning, which had nearly deprived him of life. The nerves having thus severely suffered, the muscles thereby lost their power of action; as we found the muscles of the eyes could not roll them; that the levator palpebræ superior, and occipito frontalis, were so injured, that they could neither raise the upper eyelids, nor draw up the skin of the forehead. The muscles situated about the glottis having lost their energy from the same cause, the small cartilages of the larynx became immoveable, or nearly so, which, together with the contraction of the tongue, deprived him of speech.

"Although he could make a noise, yet it was of that kind which is excited without any effect in modulating or harmonizing the voice; and a kind of whistle, which he could produce, was not shrill, but such as proceeds through a large opening in the glottis.

"This opinion of the effect of the nervous system is further confirmed by the circumstance of strong drastic purges being required to overcome the torpor to which the bowels at times were liable."

